# Association Between Trust and Mental, Social, and Physical Health Outcomes in Veterans and Active Duty Service Members With Combat-Related PTSD Symptomatology

**DOI:** 10.3389/fpsyt.2018.00408

**Published:** 2018-09-04

**Authors:** Marek S. Kopacz, Donna Ames, Harold G. Koenig

**Affiliations:** ^1^VISN 2 Center of Excellence for Suicide Prevention, U.S. Department of Veterans Affairs, Canandaigua, NY, United States; ^2^Mental Health and Chaplaincy, U.S. Department of Veterans Affairs, Durham, NC, United States; ^3^VA Greater Los Angeles Healthcare System, Los Angeles, CA, United States; ^4^David Geffen School of Medicine, University of California, Los Angeles Los Angeles, CA, United States; ^5^Department of Psychiatry & Behavioral Sciences, Duke University Medical Center, Durham, NC, United States; ^6^Department of Medicine, King Abdulaziz University, Jeddah, Saudi Arabia; ^7^School of Public Health, Ningxia Medical University, Yinchuan, China

**Keywords:** trust, post-traumatic stress disorder, Veterans, active duty military, depression, anxiety, pain, physical function

## Abstract

**Background:** Trust represents a complex emotion and interpersonal concept which assumes abandoning control over a given situation or set of circumstances, in turn yielding such control to another party. Advances in our knowledge of post-traumatic stress disorder and moral injury have underscored the need to more closely examine how trust stands to impact health outcomes in these disorders. The aim of the present study is to examine and identify relationships linking general trust with select health outcomes in a mixed sample of Veterans and Service members with a self-reported history of deployment to a combat theater and PTSD symptomatology.

**Methods:** This study applied a cross-sectional methodology, surveying *n* = 427 participants recruited across six sites. This included 373 Veterans and 54 active duty Service members in the United States. Measures included demographic characteristics, combat exposure, general trust, post-traumatic stress disorder symptomatology, depressive/anxiety symptomatology, alcohol use, social involvement, religiosity, and physical health. Data were analyzed descriptively as well as using Pearson correlations, Student's *t*-test, and multivariate regression.

**Results:** Several significant relationships were identified, indicating an inverse relationship between trust and PTSD, depressive, and anxiety symptomatology. Greater levels of trust were also significantly associated with increased social interaction and religiosity. Lastly, no significant associations were identified with either physical functioning or pain level.

**Conclusion:** The findings suggest that trust is correlated with a variety of health outcomes in Veterans and Service members affected by combat-related PTSD. Additional, hypothesis-driven research, informed by longitudinal data, is needed to better understand how trust stands to impact health outcomes, including the development of strategies and intervention options for repairing trust.

## Introduction

Developing and establishing trusting relationships remains essential to healthy human development. Trust represents a complex emotion and interpersonal concept which assumes abandoning control over a given situation or set of circumstances, in turn yielding such control to another party (1). An enhanced or diminished capacity for trust stands to tangibly impact individual well-being across the biological-psychological-social spectrum (2). Of note, however, is that only a limited literature has informed understandings of how disturbed trust stands to impact the health and well-being of Veteran and Service member populations.

The value and importance of trust is especially well developed in the military, where the dynamic of military service pushes the issue of trust to the forefront (3–6). Operationalizing military policies or directives as well as ensuring individual and organizational safety is inherently dependent on trust between Service members to adhere to a common culture of accepted practices, principles, values, beliefs, and behaviors (7, 8). Even after military service, the presence or absence of trust remains a key factor in whether some Veterans choose to establish and maintain interpersonal as well as organizational relationships (9). For example, ensuring that Veterans and Service members retain trust in their health care providers is considered to be of critical importance. Trauma from combat experiences in particular has been significantly associated with a variety of adverse mental health outcomes (10–12).

A diminished capacity for trust usually falls under the rubric of general psychopathology and can be indicative of any number of clinical disorders. Understandings of how impacted trust stands to affect the health of Veteran and Service member populations have largely focused on samples affected by post-traumatic stress disorder (PTSD). In general terms, a diminished capacity for trust hampers access to social capital and supportive services, contributing to a downward spiral of increasing social isolation and difficulty accessing vital services (13). These understandings, however, remain limited, highlighting a need to advance our knowledge by more closely examining how trust stands to impact health outcomes in select Veteran and Service member populations.

Disturbed trust is commonly encountered in cases of PTSD (14). Disturbed trust has been cited as a reason why some Veterans do not engage in health care services (15, 16) or feel uncomfortable with available treatment options (17, 18). Disturbed trust also affects such domains as relationship functioning (19) and experiences of spirituality/religion (20). Of note is that differences have been noted in clinical presentation, pathophysiology, therapeutic responsiveness, and screening sensitivity and specificity between combat-related and non-combat-related PTSD (21–23). Such differences arguably suggest that those affected by combat-related PTSD may have unique health care needs reflective of their impacted trust. Interestingly, no published data appears to be available directly examining experiences of trust, or any health implications thereof, in populations specifically affected by combat-related PTSD. Depending on the study population, the prevalence of combat-related PTSD among American Veterans is thought to range from 2 to 17% (24).

An emerging body of research into moral injury (MI) has also informed understandings of trust among Veterans and Service members. There is presently no clinical threshold or diagnostic standard to identify cases of MI. Further, there is no single, standardized definition of MI which would extend across clinical-therapeutic settings (25). Still, MI is recognized as a focus of clinical concern, conceptually and clinically distinct from PTSD (26). One frequently cited definition of MI is that of “a deep sense of transgression including feelings of shame, grief, meaninglessness, and remorse from having violated core moral beliefs” (27). Such transgressions occur in the context of potentially morally injurious events (e.g., violence, human carnage, painful loss, feelings of betrayal by one's leaders) (28–31). Compared to PTSD, the impact of MI on trust is thought to be much greater. Among those affected by MI, the capacity for trust is believed to be lost, impaired, or even destroyed (29, 32), leaving Veterans and Service members susceptible to an expectancy of harm, exploitation, and humiliation from others (33). No published prevalence estimates of MI are available, though combat Veterans have been found to have a high intensity of exposure to potentially morally injurious events (34). In some cases, PTSD and MI may also present as co-morbidities (26).

The aim of the present study is to examine and identify relationships linking general trust with select psychological, social, religious, and physical health outcomes in a mixed sample of Veterans and Service members in the United States. This study is unique in its use of a sample with a history of deployment to a combat zone as well as PTSD symptomatology. The present study adds to the extant literature by examining bivariate and multivariate relationships involving general trust, affording a more robust understanding of how trust stands to impact the health and well-being of Veterans and Service members with combat-related PTSD symptomatology. The findings could serve to inform future research aimed at developing interpersonal as well as organizational trust among combat Veterans and Service members, in addition to mitigating any adverse health effects resulting from having difficulty with general trust.

## Methodology

Participants for this cross-sectional study were recruited from six different sites. This included a sample of *n* = 373 Veterans recruited from the Department of Veterans Affairs (VA) Medical Center (MC) in Durham (*n* = 72; North Carolina), VA Greater Los Angeles Healthcare System (*n* = 99; California), Charlie Norwood VAMC (*n* = 119; Augusta, Georgia), Michael E. DeBakey VAMC (*n* = 48; Houston, Texas), Audie L. Murphy VAMC (*n* = 35; San Antonio, Texas). A sample of *n* = 54 active duty Service members were recruited through Liberty University (*n* = 54; Lynchburg, Virginia). Only Veteran or active duty Service members, with a self-reported history of deployment to a combat theater, and exhibiting PTSD symptoms were included in this study.

The data analyzed here were drawn from a larger study examining the psychometric properties of a measure of moral injury. A detailed methodology of this larger study has been published elsewhere (35). In brief, after informed consent was obtained, paper questionnaires were completed in person at all sites except the Liberty University site where the questionnaire was completed online. Participants were compensated with a $25 gift card for their time. This study was approved by the institutional review boards (IRBs) and Research & Development (R&D) Committees at Duke University as well as at each data collection site. The demographic, military, social, religious, psychological, and physical health characteristics of the sample are presented in Table 1.

**Table 1:** Sample characteristics and bivariate associations between trust (GTS) and demographic, psychological, social, and physical health outcomes.

	**Mean (SD)/% (n)**	**Trust (r or t)**
**DEMOGRAPHIC**		
Age, years	53.6 (14.7)	*r* = 0.25^****^
Gender, % male	88.7 (377)	*t* = 1.1
Race, % Caucasian	39.2 (165)	*t* = 2.0^*^
Education, years	14.1 (3.3)	*r* = 0.10^*^
Marital status, % married	49.2 (207)	*t* = 1.2
**MILITARY**		
Combat, % involved	69.3 (293)	*t* = 0.5
Combat theater, % Middle East	54.1 (229)	*t* = −3.6^***^
Time since deployed, years	23.0 (18.2)	*r* = 0.16^**^
**SOCIAL**		
Relationship quality (range 1–10)	6.4 (2.6)	*r* = 0.39^****^
Community involvement (range 1–10)	3.9 (2.6)	*r* = 0.32^****^
**RELIGIOUS**		
Christian affiliation, % Christian	82.8 (351)	*t* = −0.9
Religious commitment (BIAC) (10–100)	43.9 (20.9)	*r* = 0.15^**^
**PSYCHOLOGICAL**		
PTSD diagnosis (self-reported; % yes)	81.3 (340)	*t* = −2.6^*^
PTSD severity (PCL-5; 0–80)	52.3 (16.2)	*r* = −0.20^****^
Depressive symptoms (HADS; range 7–28)	16.6 (4.1)	*r* = −0.36^****^
Anxiety symptoms (HADS; range 7–28)	19.5 (4.1)	*r* = −0.33^****^
Alcohol use, % more than 2 drinks/day	11.1 (47)	*t* = 0.2
**PHYSICAL**		
Pain severity (range 1–10)	6.0 (2.6)	*r* = −0.08
Physical impairment (range 1–10)	5.7 (2.8)	*r* = −0.05

* *p < 0.05*,

** *p < 0.01*,

*** *p < 0.001*,

**** *p < 0.0001*.

*GTS, 6-item General Trust Scale; PCL-5, PTSD Checklist–DSM5 Military Version; HADS, Hospital Anxiety and Depression Scale*.

We applied several procedural remedies in an effort to mitigate any potential for common method bias (36). As part of the informed consent process, the sample was duly informed that responses would not be applied for diagnostic purposes nor would responses come to bear on the Veteran's or the Service member's provision of health care services or other benefits. Further, all responses were provided anonymously. The survey packet included a variety of questions and instruments with instructions designed to preclude any issues related to question order or “socially desirable responses.” Lastly, our measurements were in large part limited to high-quality empirically validated and published instruments which have already been extensively used in research.

### Measures

#### Demographic characteristics

Respondents were asked their age, gender, race, education, and marital status. Respondents were also asked their religious affiliation, with the following answer options: Christian, Jewish, Hindu, Muslim, Buddhist, other, no affiliation, and atheist/agnostic.

#### General trust

The 6-item General Trust Scale (GTS) was used to assess beliefs about the honesty and trustworthiness of others (37). The GTS has been extensively used in studies examining general trust (38–40). The original validation study provided Cronbach's alpha (α) values of 0.72 in a sample of American college students, 0.78 in an American general population sample, 0.76 in a sample of Japanese students, and 0.70 in a Japanese general population sample. In the present sample, α = 0.85. This is the first known study to apply the GTS in a mixed sample of Veterans and active duty Service members. Factor structure and across-sample correlations of factor loadings were generally high. For the purposes of the present study, GTS response categories were expanded from a 5-point to a 10-point Likert-type scale, yielding a total composite GTS score range of 6–60, with higher scores indicative of greater trust. Principle components factor analysis of the GTS in the present study demonstrated a single factor explaining >90% of the variance in the GTS.

#### PTSD

The PTSD Checklist for DSM-5 (PCL-5) is a 20-item measure assessing for the symptoms required for a PTSD diagnosis per criteria outlined in the *Diagnostic and Statistical Manual, Fifth Edition* (41, 42). The PCL-5 has shown high reliability and strong associations with combat exposure and functional impairment in military personnel (43, 44). Scores on the PCL-5 above a cutoff of 31–33 are reported to have the highest quality of efficiency in determining a DSM-5 diagnosis of PTSD (sensitivity of 0.88, specificity of 0.69, and positive predictive value of 0.81). In the present sample, α = 0.94. Participants were additionally asked if they had ever received a formal clinical diagnosis of PTSD (yes or no).

#### Combat-related symptomatology

All respondents self-reported either (a) deployment to a combat zone, without combat involvement or (b) deployment and combat involvement. For the purposes of the present study, this is taken to be indicative of combat-related symptomatology. A variety of deployment-related stressors have been associated with adverse mental health outcomes (45–47). Respondents were also asked their theater(s) of combat (e.g., Middle East, Vietnam, Korea, WWII, etc.) and the number of years since their last deployment.

#### Depressive/anxiety symptomatology

The 14-item Hospital Anxiety and Depression Scale (HADS) assesses for anxiety and depressive symptoms, each measured by seven items (48). The HADS has been reported to have high internal reliability (α = 0.85 for the anxiety subscale, α = 0.84 for the depression subscale, and α = 0.89 for the overall scale) (49). In the present sample, α = 0.86.

#### Alcohol use

Daily alcohol intake was measured using a single item on a 4-point scale, ranging from “none” to “a lot (>6 drinks/day)”. For the purposes of data analysis, responses were dichotomized into (a) < 2 drinks/day and (b) >2 drinks/day.

#### Social involvement

Respondents were asked to respond to two questions asking about (a) the quality of their relationships with spouse, children, and friends and (b) their level of involvement in community activities (other than religious group participation). Each was rated on a scale from 1 (not good/not at all) to 10 (very good/a great deal). The scores on the two items were summed to create a composite score ranging from 2 to 20, where higher scores are indicative of greater social involvement. In the present sample, α = 0.57.

#### Religiosity

The 10-item Belief into Action Scale (BIAC) is used to assess religious involvement (50, 51). This measure assesses degree of religious commitment, time spent in religious activity, and money given for religious causes. Each item is scored on a scale from 1 to 10, yielding a composite score range of 10–100, with higher scores indicating greater religiosity. In the original validation study, the internal reliability (α = 0.89, 95% *CI* = 0.86–0.91) and test-retest reliability for the BIAC (intra-class correlation or ICC = 0.92, 95% *CI* = 0.87–0.95) were high. The scale has robust convergent, discriminant, and factor analytic validity (one factor explaining 94% of variance). In the present study, α = 0.90.

#### Physical health

Difficulty engaging in physical activity level was assessed with a single item rated on a 0 to 10 scale (0 = no difficulty with physical activity, 10 = great difficulty with physical activity). Current pain level was also assessed with a single question (“How much physical pain do you have on a daily basis?”) likewise with ratings from 0 to 10 (0 = no pain, 10 = severe pain).

#### Missing values

If more than 50% of responses were left unanswered on the GTS, then such individual cases were removed from data analysis by list-wise deletion. In cases of missing items, if participants answered at least 50% of items on a given scale, the average of items answered was substituted for the missing item value. Missing values had to be substituted in 2.4% of GTS cases (10 cases; nine involving a substitution of one item and one case involving two items), 9.9% of PCL-5 cases, 8.3% of HADS cases, < 0.5% of the social involvement questions, and 5.9% of BIAC cases.

### Statistical analyses

Means (standard deviations) and frequency distributions were calculated to describe the sample. Associations between trust (6-item GTS) and demographic, military, social, religious, psychological, and physical health characteristics were examined using Pearson correlations for bivariate analysis of continuous variables and the Student's *t*-test for comparison of trust scores across dichotomized categorical variables. Multivariate regression was used to examine the association between trust and mental, social-religious, and physical health states, controlling for demographic and military characteristics. First, all demographic and military characteristics were included in full multivariate models; second, only characteristics associated with the outcomes at *p* < 0.20 were included in final reduced models. Statistical significance was set at *p* < 0.05. SAS (version 9.3; SAS Institute Inc., Cary, North Carolina) was used for all analyses.

## Results

**Figure 1:**
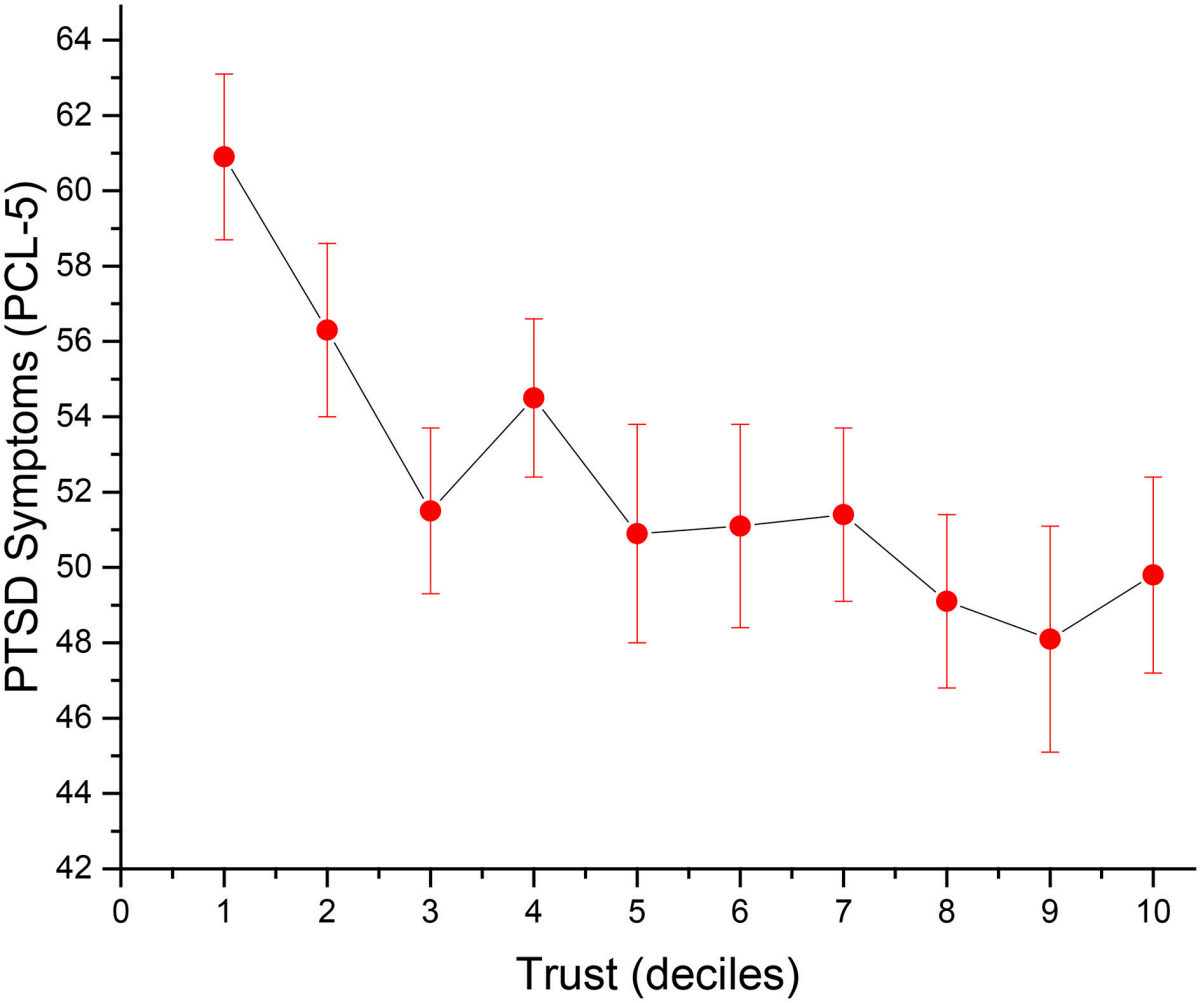
Relationship between trust and PTSD symptoms (standard errors) (uncontrolled).

A total of *n* = 7 (1.6%) individual cases were removed from data analysis owing to the omission of more than 50% of items on the GTS. The mean on the GTS was 35.1 (*SD* = 11.0) ranging from 6.0 to 54.0, with a median of 36.0 (*n* = 420). No significant difference on trust was found between Veterans and Active Duty Military on GTS scores (35.0, *SD* = 11.1, vs. 35.5, *SD* = 9.9, respectively). Those who were older, white Caucasian, more educated, deployed to combat theaters other than the Middle East (i.e., Vietnam, etc.), and deployed longer ago, all had higher trust scores (Table 1). With regard to social interactions, Veterans and Active Duty Military who scored higher on the GTS reported greater community involvement and better relationships with family and friends, and were significantly more religious as well. Greater trust was also associated with a lower likelihood of self-reporting having received a formal PTSD diagnosis and less severe PTSD symptomatology (*r* = −0.20, *p* < 0.0001), as well as less depression and lower levels of anxiety (Figure 1). Trust was not associated with alcohol intake, nor was it significantly related to either daily pain severity or impairments in physical functioning.

### Multivariate analyses

The relationships between trust and psychological, social, religious, and physical health were examined in multivariate regression models, controlling for demographic and military characteristics (Table 2).

**Table 2:** Multivariate associations between trust, psychological, social, and physical health outcomes.

	**PTSD *B (SE)***	**Depression *B (SE)***	**Anxiety *B (SE)***	**Social *B (SE)***	**Religiosity *B (SE)***	**Pain *B (SE)***	**Physical functioning *B (SE)***
Age, years	—	—	−0.04 (0.02)	—	0.04 (0.07)	0.03 (0.01)^***^	0.03 (0.01)^**^
Gender, female	—	—	—	—	—	—	—
Race, Caucasian	−5.85 (1.58)^***^	—	—	—	−5.92 (1.94)^**^	−1.19 (0.26)^****^	−1.06 (0.28)^***^
Education, years	—	−0.12 (0.06)^*^	—	0.15 (0.06)^*^	0.82 (0.29)^**^	−0.06 (0.04)	−0.07 (0.04)
Marital status, married	—	1.08 (0.38)^**^	0.69 (0.38)	—	—	—	—
Christian (yes)	—	−0.59 (0.49)	—	—	18.9 (2.5)^****^	—	—
Combat involved (yes)	—	−0.75 (0.40)	—	1.14 (0.42)^**^	—	—	0.41 (0.29)
Combat theater (ME)	0.32 (1.57)	—	0.37 (0.58)	−0.87 (0.40)^*^	—	—	—
Time since deployed (years)	—	—	—	—	—	—	—
Trust (6-item GTS)	−0.27 (0.07)^***^	−0.14 (0.02)^****^	−0.11 (0.02)^****^	0.15 (0.02)^****^	0.25 (0.09)^**^	−0.02 (0.01)	−0.01 (0.01)
Model R-square (n)	0.07**** (410)	0.17**** (403)	0.14**** (409)	0.20**** (404)	0.18**** (407)	0.10**** (410)	0.07**** (407)

* *p < 0.05*,

** *p < 0.01*,

*** *p < 0.001*,

**** *p < 0.0001*;

*ME, Middle East; GTS, General Trust Scale.Only variables associated with outcome at p < 0.20 in full models included in final models above*.

#### PTSD symptomatology

Among demographic and military factors in full models that included all of these characteristics, only two were related to PTSD symptom severity (at *p* < 0.20). Being non-White (i.e., Black or Hispanic, primarily) was associated with greater PTSD severity, as was a history of deployment to the Middle East combat theater. When controlling for both of these factors, however, trust remained strongly and inversely associated with PTSD symptoms (*B* = −0.27, *SE* = 0.07, *p* = 0.0002).

#### Depressive symptomatology

In the full model, greater depressive symptoms were associated with less education, being married, not being Christian, and not being actively involved in combat. Reduced models controlling for these demographic and military factors indicated that greater trust remained inversely related to depressive symptoms and was the strongest of all correlates (*B* = −0.14, *SE* = 0.02, *p* < 0.0001).

#### Anxiety

In the full model, greater anxiety was respectively reported by younger participants, married respondents, and those deployed to the Middle East. Controlling for these factors, greater trust remained inversely related to anxiety symptoms, and again, was the strongest and only significant inverse correlate (*B* = −0.11, *SE* = 0.02, *p* < 0.0001).

#### Social interaction

In the full model, social interaction was greater among those with more education, those who were actually involved in combat, and those who were not deployed to the Middle East (i.e., those indicating they served in Vietnam, Korea, World War II, or other theaters). Again, greater trust remained significantly related to, and was the strongest predictor for, greater social interaction, even after controlling for these factors (*B* = 0.15, *SE* = 0.02, *p* < 0.0001).

#### Religiosity

In the full model, those who were older, non-White, with more education, and Christian reported higher scores on religious involvement. After controlling for these factors in the reduced model, greater trust remained significantly correlated with greater religiosity (*B* = 0.24, *SE* = 0.09, *p* = 0.005).

#### Physical health

Daily pain severity was related to older age and less education, most strongly in non-White race respondents, but was not related to level of trust in either the full model or the reduced model. Likewise, impairments in physical functioning were related to older age, less education, involvement in actual combat, most strongly in non-White race respondents, but was again unrelated to level of trust.

## Discussion

The aim of the present study was to examine the relationship between general trust and select health outcomes, controlling for potentially confounding variables, in a population of Veterans and Service members with combat-related PTSD symptomatology. Several significant relationships were identified, indicating an inverse relationship between trust and PTSD, depressive, and anxiety symptomatology. Greater levels of trust were also significantly associated with increased social interaction and religiosity. Lastly, no significant associations were identified with either physical functioning or pain level. To the best knowledge of the authors, these findings appear to be without precedent in the literature, underscoring a need for additional, hypothesis-driven research.

The present findings highlight how general trust is correlated with a variety of health outcomes in a sample of Veterans and Service members with combat-related PTSD symptomatology. As Service members continue to return from foreign theaters of combat and return back into the community as Veterans, developing understandings of the clinical importance of general trust will no doubt remain a focus of empirical attention. Irrespective of clinical condition, enhanced or diminished trust among Veterans and Service members has also been found to impact such domains as suicide risk screenings (52), employment (53), relationships (54), and psychosocial readjustment (55–57). Future research should invariably include a focus on identifying viable options and avenues for facilitating trust among those affected by combat-related PTSD symptomatology. At present, (re)establishing the capacity to trust is described as a secondary outcome of existing PTSD treatment options, with cognitive behavioral therapy being the most promising treatment for facilitating general trust (58–60).

In the present sample, greater levels of trust were associated with both increased social interaction and greater religiosity. The implications of this finding potentially extend beyond combat-related PTSD symptomatology and may also serve to inform an emerging body of research into MI. For example, issues related to religion and spirituality have been posited as potential “root causes” of MI (25, 61). The negative affect encapsulated by MI may draw from faith-based standards of moral conduct violated in the course of a morally injurious event. Through social interaction (e.g., religious practice), those affected by MI are exposed to different sources of social capital that might help them rebuild trust (62). In the cases of both PTSD as well as MI, higher levels of trust intuitively suggest a salutary cycle of support, such as a propensity to engage with different sources of support, reinforcing and developing existing general trust, ultimately supporting favorable therapeutic outcomes.

Trust is dependent on a variety of factors. One might reasonably argue that some Veterans may have also had trust issues preceding their military service. For this reason, future research should also be guided by longitudinal data, including such variables as history of relationships with family of origin, any experiences of abandonment (e.g., “broken home,” foster care), relationships with significant others, and marital history. It is not uncommon for Veterans to have difficult pre-military family experiences (63). Enlistment in the military services may, in some cases, be motivated by the desire for an alternative, more trustworthy “family experience” (64). In the United States, the issue of qualifying for Department of Veterans Affairs (VA) benefits also stands to tangibly impact general trust in this organization, with implications extending far beyond just populations affected by combat-related PTSD. In recent years, trust between Veterans and the VA health care system has been complicated by organizational issues and challenges (65–67). Another avenue for future research might include comparatively examining levels of general trust, inclusive of any associated health effects, among Veterans who have qualified for VA services vs. those who did not qualify.

The generalizability of the findings reported here and their interpretation is limited by several factors. This was a sample of convenience that involved volunteers who agreed to participate. As a cross-sectional study, it was impossible to determine causality (e.g., whether greater trust led to less PTSD, depression, anxiety, and better social relationships, or vice versa). Participants were recruited from sites located primarily in the southern United States, so these results may not apply to Veterans and/or Service members more generally, nor do they take into consideration certain regional, cultural, or contextual influences which may not be present in other parts of the country. Future research should consider diversifying sample recruitment across multiple military, civilian, and geographic regions/settings. The dynamic governing trust among Service members is presumably different from that of Veterans. Any such bias would have been mitigated by the inclusion of only a small subsample of active duty Service members (*n* = 54; 13% of the sample). The present study did not assess for different types of trauma experienced by the sample. Lastly, all data was self-reported and not verified by official government and/or clinical records.

Notwithstanding these limitations, the findings of this cross-sectional study provide an important degree of insight into the association between general trust and select health outcomes in a sample of Veterans and Service members with PTSD symptomatology. Understandings of how trust impacts health outcomes remain limited. Further, a paucity of evidence-based support options exist for building trust (68, 69). By drawing attention to the possibility that increasing trust may lead to more favorable health outcomes, the intention was to inform future research into trust-building clinical interventions. The strength of these findings is reinforced by the use of a large, multi-site sample inclusive of both Veterans and Service members with PTSD symptomatology, the use of psychometrically validated measures, and the careful assessment and control for numerous demographic and military characteristics. Future research should consider longitudinal studies of trust and health outcomes, developing comparative studies between combat- and non-combat-related PTSD, and seeking to better understand the role of faith in the development of trust.

## Conclusion

This cross-sectional study sought to examine the relationship between general trust and select health outcomes in a mixed sample of Veterans and Service members with PTSD symptomatology. The findings suggest that trust is correlated with a variety of health outcomes in this group. Several significant relationships were identified between trust and clinical symptomatology of PTSD, depression, and anxiety, respectively. Trust was also associated with social interaction and religiosity. The findings suggest several avenues for additional research into how disturbed general trust impacts the health of Veterans and Service members with PTSD.

## Ethics statement

This study was conducted in accordance with IRB and R&D Committee approval at Duke University as well as each data collection site. Prior to taking part, all participants gave written informed consent in accordance with the latest version of the Declaration of Helsinki.

## Author contributions

MK, DA, and HK: Contributed to writing article. HK: Data collection, database maintenance, data analysis.

### Conflict of interest statement

The authors declare that the research was conducted in the absence of any commercial or financial relationships that could be construed as a potential conflict of interest.
